# Self-Powered Microsystem for Ultra-Fast Crash Detection via Prestressed Triboelectric Sensing

**DOI:** 10.34133/research.0753

**Published:** 2025-07-02

**Authors:** Yiqun Wang, Yuhan Wang, Xinzhi Liu, Xiaofeng Wang, Keren Dai, Zheng You

**Affiliations:** ^1^ Department of Precision Instrument, Tsinghua University, Beijing 100084, PR China.; ^2^ Beijing Advanced Innovation Center for Integrated Circuits, Tsinghua University, Beijing 100084, PR China.; ^3^ School of Mechanical Engineering, Nanjing University of Science and Technology, Nanjing 210094, PR China.

## Abstract

Reliable detection of high-*g* shocks in extreme impact scenarios, such as automobile collisions, is essential for ensuring occupant safety. Conventional shock sensors based on piezoresistive or capacitive mechanisms often underperform in high-*g* environments due to their structural complexity, resulting in delayed or missed detection. Here, we present a self-powered high-*g* shock sensor that combines a triboelectric transducer with a prestressed structure to deliver large signal amplitude and minimal oscillation. The prestress mechanism enhances initial contact strength, achieving a 400% increase in signal amplitude and reduced oscillation. We further developed a self-powered, compact (<4.5 cm^3^) microsystem that integrates the shock sensor, a signal processing module, airbag triggering circuitry, and a high-*g*-resistant supercapacitor as a backup power source. The microsystem achieves ultra-fast shock detection and airbag activation with a delay of less than 0.2 ms. Furthermore, its power demand is 80% lower than that of commercial high-*g* sensors, while the pre-charged supercapacitor ensures operational stability. To further extend the functionality of the device, we designed a lightweight collision target classification algorithm using ensemble learning and feature importance analysis, which could accurately distinguish between automotive collisions with hard, brittle, and soft materials. This study advances triboelectric nanogenerators for high-*g* shock sensing, offering improved reliability, performance, and real-world adaptability.

## Introduction

Detecting high gravitational acceleration (high-*g*) impacts presents a critical challenge owing to the detrimental effects of extreme mechanical shock [1,2]. In key sectors such as the defense and automotive industries, such impacts are tied to safety-critical actions like detonator activation or airbag deployment, both of which are crucial for protecting human lives. Consequently, impact sensors must respond reliably and effectively to transient shocks that exceed 1,000 × *g*, and in some cases, even 10,000 × *g*. Traditional shock sensors, typically based on piezoresistive or capacitive principles [3–5], often fail under these extreme conditions due to the complexity of their mechanical structures [6]. These failures can cause erroneous detonations or airbag malfunctions, threatening safety. To overcome these limitations, there is an urgent need for shock sensors with simple structures, self-powered operation, independent signal generation [7], and strong signals.

Triboelectric nanogenerators (TENGs) [8–12] enable self-powered motion [13–19] and mechanical sensing [18,20–25], with recent advances extending into shock detection. Dai et al. [26] designed a TENG-based accelerometer using aluminum/polydimethylsiloxane-copper electrodes that bend under high-*g* impacts to generate signals. Garcia et al. [27] developed a polyvinylidene fluoride/polyvinyl pyrrolidone nanofiber sensor that produces a voltage that increases linearly with shock energy. They later extended this to a dual-functional sensor that also monitored impact velocity via electrode separation [28]. Other multimodal approaches include Alzgool et al.’s [29] TENG/micro-electro-mechanical system (MEMS) hybrid detector, where contact between aluminum and polyimide surfaces activates a MEMS-based switch, and Lu et al.’s [30] magnet-assisted accelerometer using sliding electrodes. Despite these advances, several challenges remain, including weak signal amplitude, undefined triggering thresholds, and chaotic oscillations that require complex signal conditioning circuits and compromise shock resistance. Streamlined designs with enhanced signal integrity and better system integration are essential for practical applications.

In this study, we present a triboelectric high-*g* shock sensor that delivers large signal amplitude with minimal oscillation. This is achieved through a prestress mechanism in which a silicone elastomer is compressed during sensor fabrication. Compression enhances the initial contact strength between the triboelectric layer and electrode surface, resulting in stronger signals with reduced oscillation under high-*g* impacts. The prestressed structure also establishes an impact threshold that prevents false triggering. To address the limitations of previous theoretical models, which have primarily focused on changes in spatial potential distribution, we developed a comprehensive multi-physics theoretical model that incorporates surface charge variation. This advancement enables complete simulation and theoretical analysis of the prestressed, self-powered shock sensor. We also established an optimization framework to maximize device performance.

We investigated the use of the sensor in 2 practical cases, namely, automotive collisions and airbag activation. First, we developed a collision scene recognition algorithm based on ensemble learning and feature importance ranking. This lightweight algorithm was designed to recognize collisions with multiple targets, including hard, brittle, and soft materials. Second, we established a compact (<4.5 cm^3^) and self-powered microsystem for practical airbag control. This microsystem integrates the sensing unit, a signal processing module, airbag triggering circuitry, and a high-*g*-resistant supercapacitor as a backup power source. Thus, it ensures rapid shock detection and low-latency airbag activation, as validated through simulated crash and airbag activation tests. This study not only expands the application scope of TENGs under extreme conditions but also introduces a novel approach for developing next-generation high-performance self-powered shock sensors.

## Results and Discussion

### Prestressed shock sensor based on triboelectric transducer

**Fig. 1.**
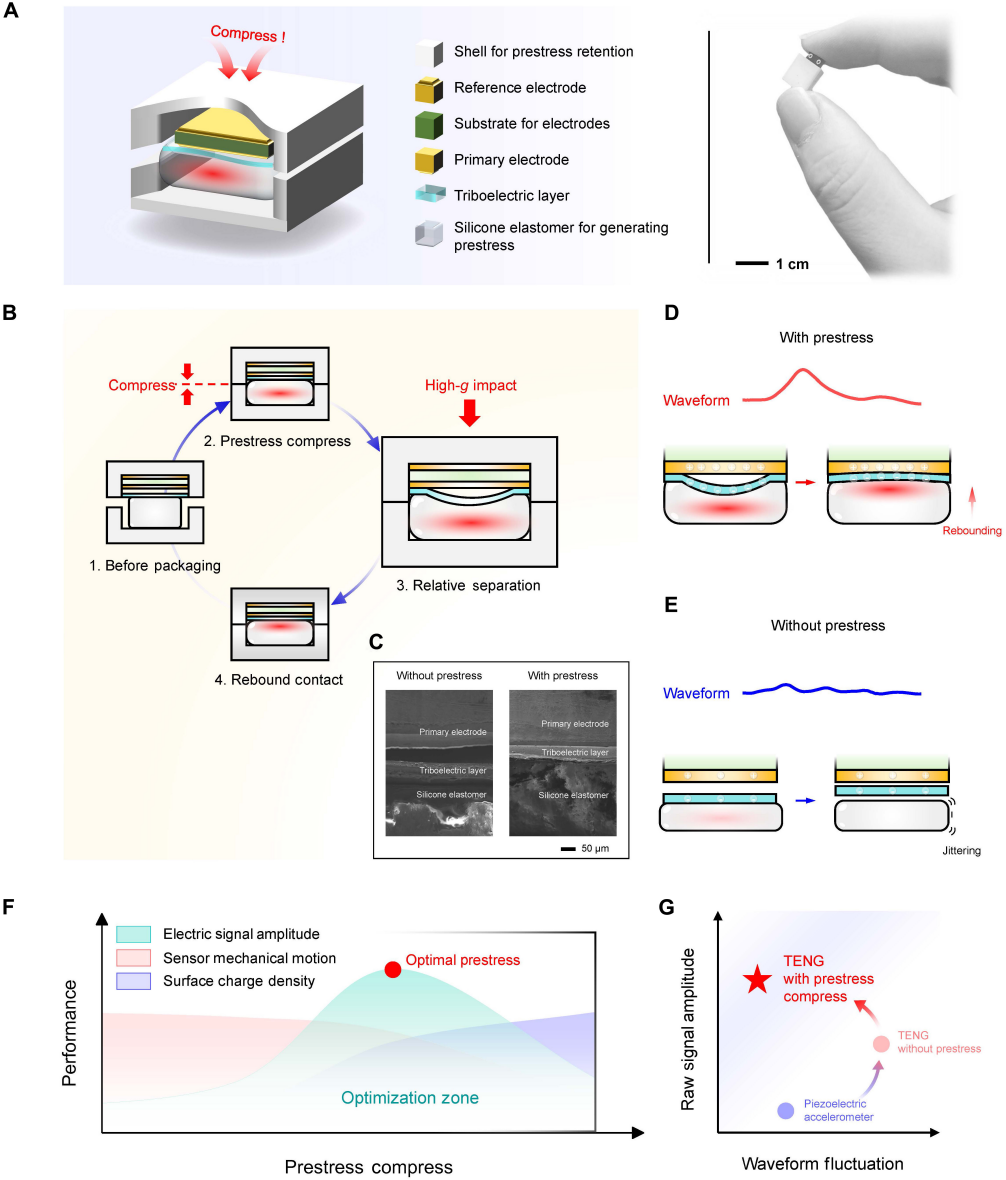
Self-powered shock sensor based on triboelectric transducer. (A) Schematic of sensor comprising housing unit to maintain prestress, reference electrode, electrode substrate, main electrode, triboelectric layer, and silicone layer for generating prestress. Prestress is applied to the silicone layer by compressing the housing. The right-hand photograph shows a prototype sensor, with dimensions of ~1 cm. (B) Working principle of the sensor. Before packaging, the sensor is in a stationary state. When prestress is applied through compression, the triboelectric layer contacts the electrode surface. Upon high-*g* shock, the triboelectric layer and electrode surface separate, generating an electrical signal. After the shock subsides, the sensor elastically rebounds to its initial state, completing one working cycle. (C) SEM images of contact interface of triboelectric generators with and without prestress. (D and E) Sensing mechanism and waveform of (D) prestressed and (E) non-prestressed sensors. The prestressed sensor produces a stable, high-amplitude waveform with enhanced signal stability, whereas the non-prestressed sensor exhibits lower signal amplitude and substantial waveform fluctuations. (F) Effect of prestress on sensor performance. Increasing the prestress enhances the signal amplitude, mechanical response, and surface charge density. Optimal performance is achieved within a specific compression range, forming an optimization zone. (G) Performance of different sensors. The prestressed triboelectric sensor demonstrates superior signal amplitude and waveform stability compared to a non-prestressed triboelectric sensor and a traditional piezoelectric accelerometer.

This study introduces a self-powered high-*g* shock sensor based on a prestressed electrostatic transducer, specifically designed for impact detection in extreme environments. Figure 1 provides an overview of the structural design, working principles, and performance optimization of the sensor.

As illustrated in Fig. 1A, the sensor is compact but with a multi-layered structure. It comprises a housing unit to maintain prestress, a reference electrode, an electrode substrate, a main electrode, a fluorinated ethylene propylene (FEP)-based triboelectric layer, and a silicone elastomer layer to generate prestress. The prestress is applied by compressing the housing during fabrication. The photograph on the right-hand side of Fig. 1A shows the prototype sensor. It has a length of approximately 1.103 cm, underscoring its miniaturized and highly integrated design.

Figure 1B details the working mechanism of the sensor. Initially, in the stationary state before packaging, no relative movement occurs between the components. Compression is applied to the housing during the packaging process to establish the prestressed state, which ensures close contact between the triboelectric layer and electrode surface. Under high-*g* impact, the sensor undergoes 2 distinct stages to convert mechanical energy into electrical signals. First, the triboelectric layer and electrode surface separate, generating an electrical signal via charge transfer. After the impact subsides, the sensor elastically rebounds to its initial state, completing one working cycle. This cyclic process enables the continuous conversion of mechanical energy into electrical signals. Furthermore, as the electrical signals are generated by the triboelectric layer during impact, the sensor functions without requiring an external power source.

The scanning electron microscopy (SEM) images in Fig. 1C illustrate the structural differences between devices with and without prestress. In the prestressed device, the primary electrode, triboelectric layer, and silicone elastomer are in tight contact and under compression. In contrast, the non-prestressed device exhibits a visible gap between the primary electrode and triboelectric layer. This demonstrates the enhanced structural integrity and functionality of the prestressed design.

The performance of the prestressed sensor is markedly superior to that of the non-prestressed sensor. As illustrated in the waveform in Fig. 1D, the prestressed sensor produces a high peak voltage following impact. The prestressed state enables the silicone elastomer to quickly return to its original position, resulting in excellent signal stability. In contrast, as shown in Fig. 1E, the non-prestressed sensor exhibits lower signal amplitude. Additionally, the signal continues to fluctuate after the impact owing to continued movement of the triboelectric and silicone elastomer layers. Thus, the tight contact between the triboelectric layer and electrode surface is essential for enhancing signal stability and sensitivity.

The mechanism by which prestress optimizes the sensor performance is shown in Fig. 1F. As the prestress applied to the silicone elastomer increases, the deformation amplitude under impact decreases monotonically, while the surface charge density increases. This inverse relationship indicates that optimization of the prestress level can maximize the sensor’s output. Under the optimal prestress, the sensor exhibits even less oscillation, highlighting the role of appropriate prestress loading in performance optimization.

Figure 1G further demonstrates the advantages of the prestressed triboelectric shock sensor. Compared to both the non-prestressed sensor and conventional high-*g* piezoelectric accelerometer, the prestressed device delivers higher signal amplitude and greater stability, with markedly reduced oscillation. For a detailed performance comparison, please refer to Table S2 and Section S17. These results confirm the reliability and effectiveness of the prestressed sensor under high-*g* shock conditions.

### Modeling, simulation, and testing of the self-powered shock sensor

**Fig. 2.**
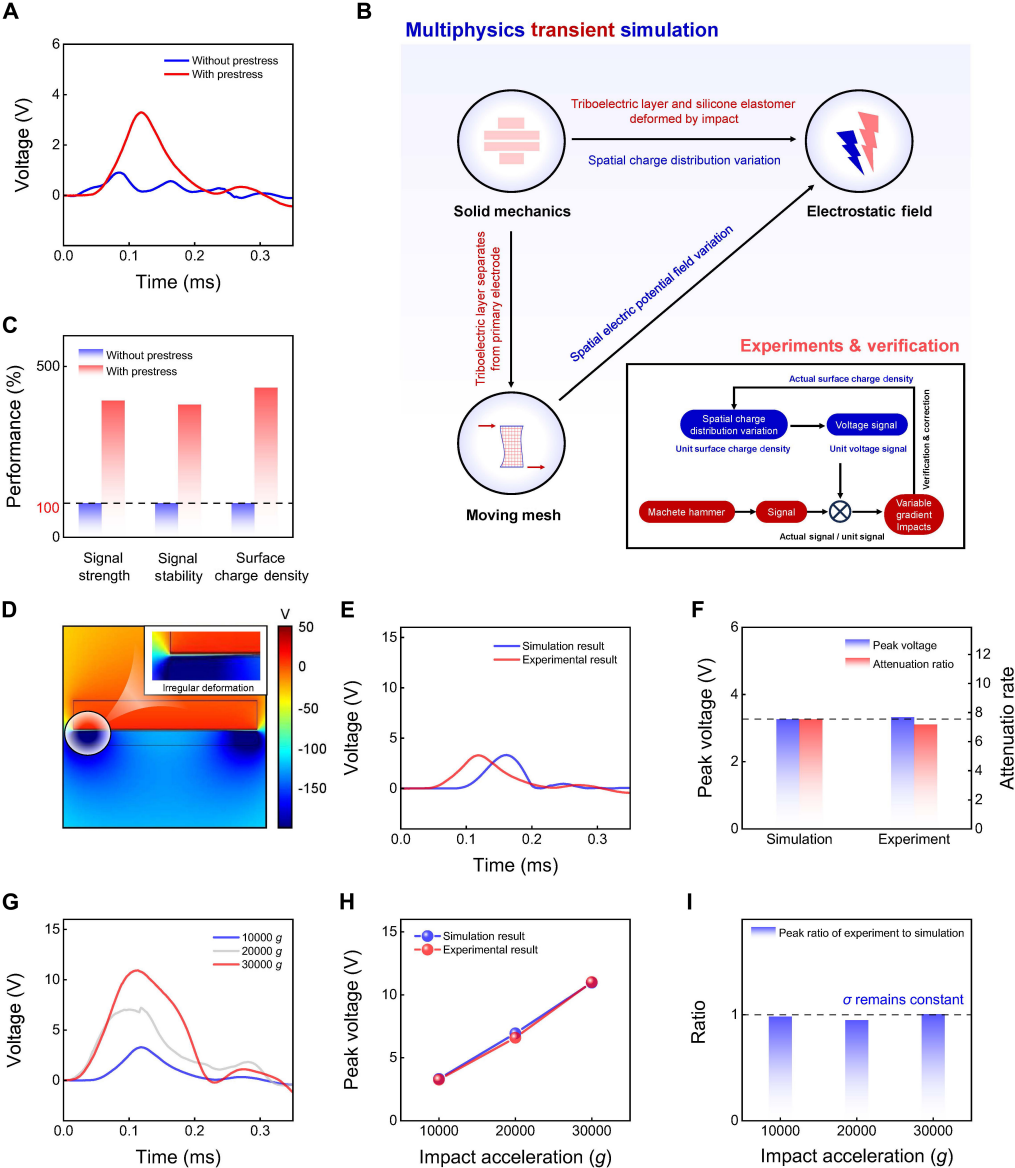
Modeling, simulation, and testing of the prestressed shock sensor. (A) Open-circuit voltage waveforms of sensors with and without prestress during impact. Applying prestress significantly increases the peak voltage and enhances signal stability. (B) Schematic of multi-physics transient simulation framework integrating solid mechanics, electric fields, and moving mesh modules to simulate the sensor’s response to impact. It elucidates the coupling mechanism of spatial charge distribution and dynamic electric field changes. The simulated and experimental spatial charge distribution, unit surface charge density, and unit charge voltage signals demonstrate strong agreement. (C) Quantitative analysis of performance improvement due to prestress. Prestress enhances the signal strength, stability, and surface charge density by more than 100% compared to the non-prestressed state. (D) Simulated transient spatial potential distribution during impact. The simulation reproduces the dynamic evolution of the electric field upon impact. (E) Simulated and experimental open-circuit voltage waveforms during impact. The high consistency between the 2 waveforms validates the accuracy of the multi-physics coupling model. (F) Simulated and experimental peak voltage and attenuation ratio. The results align closely, demonstrating the ability of the model to accurately predict the voltage output of the sensor. (G) Experimental voltage signals of the sensor under different shock accelerations (10,000 × *g*, 20,000 × *g*, and 30,000 × *g*). (H) Simulated and experimental peak voltage as a function of shock acceleration. The linear and highly consistent relationships demonstrate the excellent linearity of the sensor and the predictive capability of the model. (I) Statistical analysis of experimental-to-simulated peak voltage ratios. The consistent peak voltage ratios across different shock accelerations validate the robustness and reliability of the proposed simulation framework.

Shock testing was performed on devices with and without prestress. The open-circuit voltage signals under identical high-*g* impact conditions (10,000 × *g*) are presented in Fig. 2A. The prestressed device generates a significantly higher open-circuit voltage upon impact than the non-prestressed device, and exhibits reduced waveform oscillation. These results confirm the advantages of prestress in enhancing signal stability and reliability under extreme high-*g* impacts.

To enable accurate simulation, theoretical analysis, and optimization of the prestressed self-powered sensor, we developed a multi-physics transient theoretical model, as illustrated in Fig. 2B. This framework integrates solid mechanics with electrostatic field analysis and applies moving mesh theory to construct a time-dependent transient simulation approach. More detailed equations for multiphysics transient simulation, especially those involving solid mechanics and electrostatics, are listed in Section S1. When the sensor experiences a high-*g* impact, it undergoes rapid deformation that causes the triboelectric layer to separate from the primary electrode. This separation alters the spatial distribution of the electric potential. Simultaneously, the triboelectric layer undergoes complex, multi-modal deformation, changing the surface charge distribution. These coupled effects collectively influence the open-circuit voltage of the sensor.

Unlike conventional quasi-static simulations used for TENGs, the developed model captures the transient voltage oscillations generated during impact, as shown in Fig. S1. A more detailed comparison of the multiphysics transient simulation proposed in this work and conventional TENG simulation is shown in Table S1. To ensure model accuracy, we established a verification and calibration workflow that combines simulation with experimental testing. Using extensive experimental datasets, this workflow calibrates critical parameters within the theoretical framework, including the surface charge density. As such, it helps to reduce the gap between the simulation and experimental results.

As part of this workflow, we introduced the concept of “unit charge voltage”, defined as the output voltage generated by a device with unit triboelectric charge density under a standard impact condition (10,000 × *g*, 0.1 ms). This parameter serves as a physical reference for evaluating mechanical deformation via simulations and forms the basis for experimentally determining the surface charge density. By comparing the measured output voltage to the simulated unit charge voltage, and refining the results through repeated corrections under graded impact tests, we accurately calibrated the triboelectric surface charge density.

Incorporating the calibrated surface charge density into the simulation enabled precise prediction of key output characteristics, including the peak voltage and attenuation ratio. The peak voltage refers to the maximum amplitude of the signal waveform, while the attenuation ratio is defined as the ratio of the first to the second waveform peaks. As shown in Fig. 2C, the prestressed device exhibits >400% improvement in all 3 key metrics—peak voltage, attenuation ratio, and surface charge density—compared to the non-prestressed device.

Figure 2D shows the transient spatial electric potential distribution during impact, illustrating the dynamic evolution of the electric field. The separation between the triboelectric layer and primary electrode reveals significant but irregular deformation in the triboelectric layer (see the deformation and strain simulation in Fig. S2). This deformation leads to noticeable changes in the spatial distribution of triboelectric charge.

Figure 2E compares the voltage signals obtained from the simulations and experiments, confirming the accuracy of the theoretical model. The trends in the datasets are consistent, underscoring the reliability of the model in simulating the open-circuit voltage of the sensor. (For results of the non-prestressed device, refer to Fig. S3.) Figure 2F further compares the peak voltage and attenuation ratio between simulation and experimental results, emphasizing the consistency and predictive strength of the simulation framework.

Figure 2G presents the voltage signals of the sensor under varying shock conditions (10,000 × *g*, 20,000 × *g*, and 30,000 × *g*). The results demonstrate that stable performance was achieved under a wide range of high-*g* shock conditions. Corresponding simulation results are provided in Figs. S4 and S5. Figure 2H compares the peak voltage as a function of shock acceleration. The simulation and experimental results both demonstrated highly linear relationships. This highlights the excellent linearity of the sensor and confirms the capability of the theoretical model to accurately predict the response of the sensor under high-*g* shocks.

Finally, Fig. 2I shows the ratio of the experimental peak voltage to the simulated value for the prestressed sensor across multiple shock conditions. The ratio was consistent across varying shock conditions, thereby validating the robustness and reliability of the theoretical framework proposed in Fig. 2B. This result further confirms the stability of the proposed model in capturing the sensor’s behavior under a range of high-*g* conditions.

### Analysis and optimization of the prestress mechanism

**Fig. 3.**
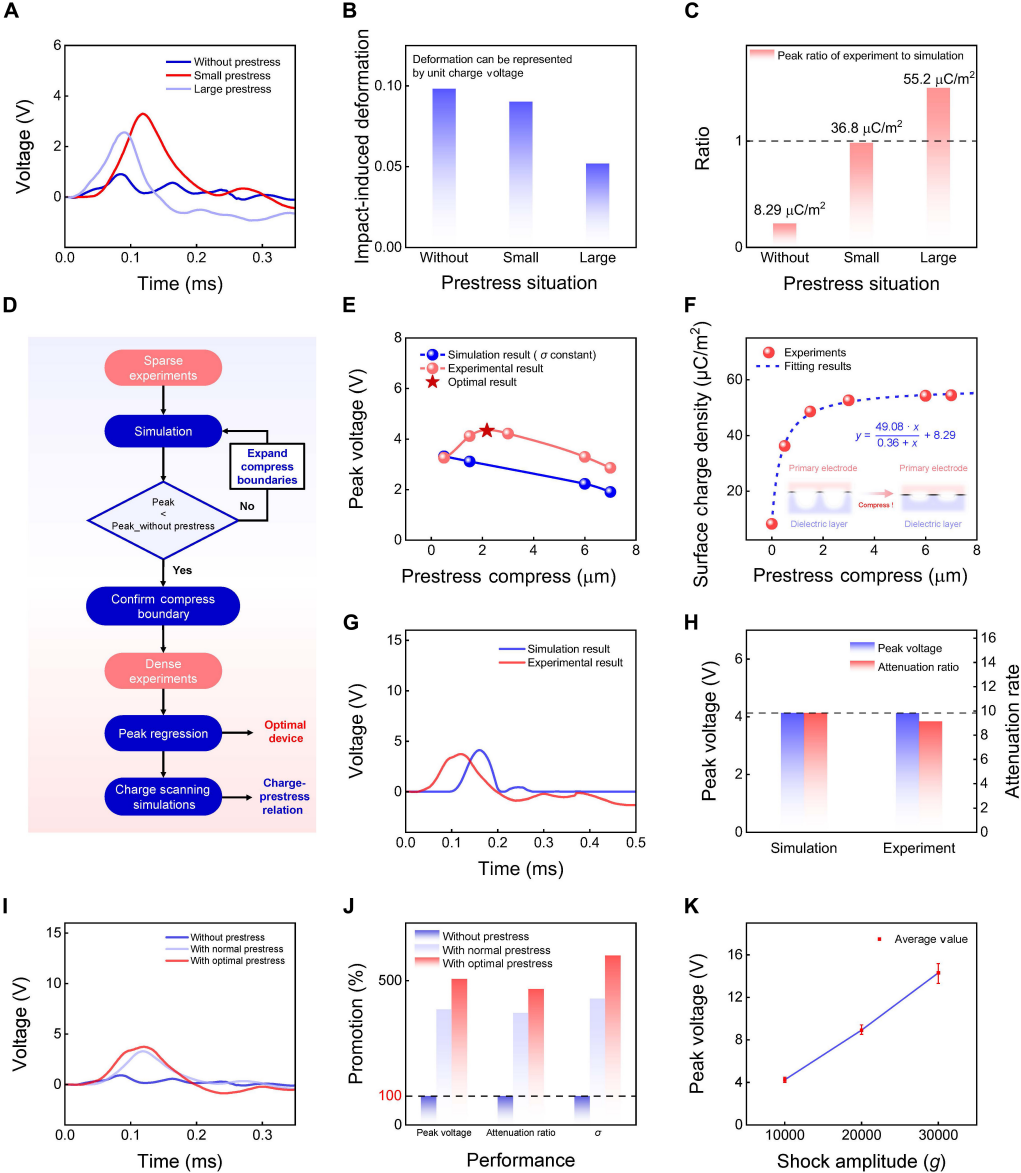
Mechanistic analysis and structural optimization of prestressed shock sensor. (A) Voltage waveforms of sensors with varying prestress conditions. As prestress increases, the peak voltage initially rises before declining at higher prestress levels. (B) Unit charge voltage for sensors with different prestress conditions, reflecting the deformation response. (C) Surface charge density for sensors with different prestress conditions, showing a steady rise from 8.29 to 55.2 μC/m^2^. (D) Workflow for prestress optimization, including experimental data extraction, peak value updating and assessment, and charge scanning with regression analysis. (E) Experimental and simulated peak voltage for sensors with different prestress conditions, with a red star indicating the theoretically optimized result. (F) Experimental data and fitting curve for prestress compression and surface charge density. (G) Simulated and experimental voltage waveforms for the optimal device, demonstrating close agreement. (H) Simulated and experimental peak voltage and attenuation ratio. The strong alignment validates the effectiveness of the optimization method. (I) Voltage waveforms for sensors with different prestress conditions, highlighting the superior performance under optimal prestress. (J) Percentage improvement in peak voltage, attenuation ratio, and surface charge density (*σ*) with conventional and optimal prestress. Optimal prestress produced remarkable enhancements. (K) Experimental peak voltage of sensor with optimal prestress under different shock accelerations, confirming a good consistency, repeatability, and linearity and performance of the sensor under high-*g* shocks.

To further optimize the performance of the prestressed sensor, we tested devices with various levels of prestress. As shown in Fig. 3A, the peak voltage initially increased with increasing prestress but began to decline after a certain point (also see the corresponding simulation results in Fig. S6). This trend suggests that there is an optimal prestress that maximizes the output voltage.

To better understand this behavior, we used the theoretical framework introduced in Fig. 2 to calculate the deformation amplitude and surface charge density of sensors with different prestress conditions. As the prestress increased, the deformation amplitude decreased (Fig. 3B), while the surface charge density steadily increased from 8.29 to 55.2 μC/m^2^ (Fig. 3C). These results align with the proposed functionality of prestress: It enhances the triboelectric charge density during shock while also limiting movement of the triboelectric layer after impact. To optimize the prestress, the increase in surface charge density must be balanced against the decrease in movement. This balance defines an optimization space for maximizing the output voltage. The results emphasize the importance of selecting an appropriate prestress level for optimizing the sensor performance (see the simulation results under different prestress conditions in Figs. S7 and S8).

To systematically identify the optimal prestress level within the defined optimization space, we developed a structured workflow, illustrated in Fig. 3D. The first step involved a set of sparse preliminary experiments using 3 devices: one without prestress, one with moderate prestress, and one with high prestress. These initial tests provided qualitative insight into the prestress mechanism and confirmed its importance as a key optimization parameter, as demonstrated in Fig. 3A to C. In the second step, we used simulations to define the range of the prestress optimization space. By gradually increasing the prestress level, we identified the boundaries of the optimization space (i.e., the points at which the output voltage no longer exceeded that of the device without prestress). The third step involved conducting detailed experiments within the optimization boundaries using devices with varying prestress levels. This was supported by regression analysis, as shown in Fig. 3E. Finally, we performed charge scanning and regression analysis to establish the relationship between prestress and surface charge density, as shown in Fig. S9.

Our analysis revealed that the surface charge density increases rapidly as the prestress increases, before gradually reaching saturation. We applied a mathematical model based on contact and friction theory (Eq. 1), which showed excellent agreement with the experimental data, as shown in Fig. 3F.

$$y=\frac{49.08x}{0.36+x}+8.29$$ (1)

Equation 1 demonstrates that increasing the prestress enlarges the true contact area at the interface between the triboelectric layer and electrode surface due to greater compression [31,32], which in turn increases the surface triboelectric charge density. However, once the prestress reaches a critical level, the contact area reaches saturation [33,34], causing the charge density to approach its upper limit. This mechanism is illustrated schematically in Fig. S10. In Eq. 1, the value “49.08” represents the maximum achievable increase in triboelectric charge density, defining the upper saturation limit (i.e., once the contact interface is fully compressed). The value “0.36” indicates the characteristic displacement that marks the critical transition point from rapid growth to saturation. Meanwhile, the value “8.29” corresponds to the initial triboelectric charge density under zero compression and serves as a baseline reference.

We then evaluated the sensing performance of the device configured with the optimal prestress, as shown in Fig. 3G to K. For this configuration, the signal waveforms predicted by the theoretical framework aligned closely with the experimental results (Fig. 3G). The simulated and experimental peak values and attenuation ratios of the sensor signals were also consistent, as shown in Fig. 3H.

Figure 3I compares voltage waveforms under 3 conditions: no prestress, conventional prestress, and optimal prestress. The results clearly show that the device with optimal prestress outperformed the other configurations. To quantify this improvement, Fig. 3J presents percentage increases in key performance metrics—peak voltage, attenuation ratio, and surface charge density (*σ*)—across the 3 conditions. The optimal prestress yielded substantial enhancements, with signal amplitude and charge density increasing by over 400% compared to the non-prestressed condition.

7optimally prestressed device under multiple shocks with 10 shocks at shock amplitudes of 10,000 × *g*, 20,000 × *g*, and 30,000 × *g*, respectively. The results confirm the sensor’s good consistency, repeatability, and linearity under high-*g* shock conditions. Furthermore, the sensor demonstrates a clear threshold effect, generating significant response voltages only when subjected to impacts exceeding 1,500 × *g*, as depicted in Figs. S11 and S12.

### Signal analysis and system integration of airbag control microsystem

The working principles and optimization of the prestressed shock sensor are thoroughly discussed in the previous sections. For practical applications, such as detecting rapid deceleration during automobile collisions to trigger airbags deployment, further improvements in signal processing and external circuitry are required. These enhancements are essential for improving the stability, user-friendliness, and functionality of the system. To address these requirements, we developed an intelligent recognition algorithm and a plug-and-play integrated microsystem specifically designed for automotive collision detection and airbag control, a critical application scenario with significant demand. This integrated system is designed to operate seamlessly with the prestressed shock sensor and supports reliable airbag deployment.

#### Analysis and identification of complex shock conditions

In real-world automotive collisions, airbag deployment systems must not only measure the intensity of impact but also distinguish between different collision scenarios. This capability is crucial to avoid both under-deployment and false deployment of airbags while also informing broader decision-making in vehicle control systems. For instance, collisions with hard objects like steel fences produce strong shocks that require immediate airbag activation to protect the driver and passengers. Conversely, impacts involving brittle materials such as glass windows or curtain walls may result in material breakage without severe deceleration, while collisions with softer structures such as soil may cause the vehicle to gradually decelerate. If the impact is not severe enough to warrant airbag deployment, airbag activation should be avoided to prevent injury. However, immediate braking is still required to mitigate secondary hazards such as tire damage, further collisions, or the vehicle becoming immobilized in soil.

To address this diverse range of scenarios, we analyzed the response of the sensor to impacts with different collision targets, including steel, glass, and clay soil. Using the collected data, we developed a simple, effective, and easily deployable recognition algorithm based on machine learning and feature importance ranking. This algorithm enables the sensor to identify the collision conditions, thereby improving both safety and decision-making in automotive control systems.

**Fig. 4.**
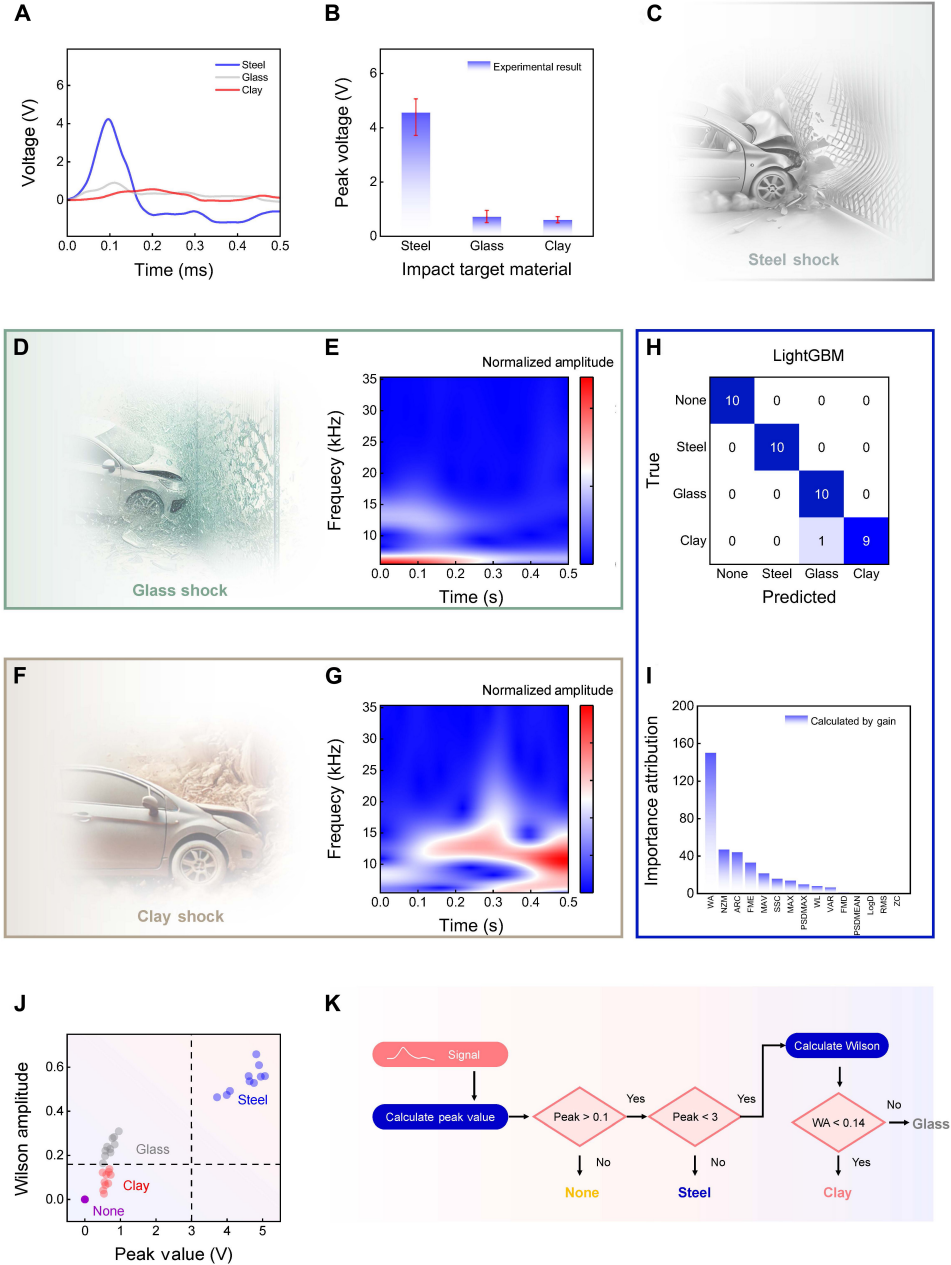
Signal analysis and recognition of different impact targets using the prestressed shock sensor. (A) Voltage output for impacts with steel, glass, and clay soil. The signals exhibit noticeable differences, particularly in terms of peak voltage and signal duration. (B) Peak voltage for different impact targets. The peak voltage for steel is significantly higher than that for glass and clay, while the peak voltages for glass and clay are relatively similar. (C) Schematic of car colliding with steel fence. (D) Schematic of car colliding with glass curtain wall. (E) Time–frequency analysis of signals generated during impact with glass using wavelet transform. (F) Schematic of car colliding with clay soil. (G) Time–frequency analysis of signals generated during impact with clay soil using wavelet transform. The glass impact signal contains higher-frequency components, whereas the clay soil impact signal is dominated by low-frequency characteristics. (H) Confusion matrix of classification results for 4 impact conditions: none, steel, glass, and clay soil. Classification was performed with high accuracy using the LightGBM machine learning algorithm. (I) Feature importance ranking in the LightGBM model. Gain calculations identified peak voltage and Wilson amplitude (WA) as the most influential features for classification. (J) Two-dimensional feature distribution plot of peak voltage and Wilson amplitude. Different impact conditions form distinct regions within this feature space, enhancing classification and recognition. (K) Decision flowchart for impact condition recognition, utilizing peak voltage and Wilson amplitude as primary indicators. The flowchart outlines step-by-step decision rules for accurately identifying impact conditions.

Figure 4A presents the voltage output signals for impacts with steel, glass, and clay soil. The signals display clear differences, particularly in terms of the peak voltage and signal duration. Impacts with steel, such as when a car collides with a steel fence (Fig. 4C), generate much higher peak voltages than collisions with glass and clay soil (Fig. 4D and F, respectively), enabling collisions with steel to be easily distinguished. In contrast, impacts with glass and clay soil produce more similar peak voltages, as shown in Fig. 4B, hindering differentiation. However, the voltage outputs still differ in terms of waveform shape and duration. This highlights the need to analyze additional signal features to effectively identify these collision targets.

Further analysis of the impact signals from glass and clay soil was performed using wavelet transform, as shown in Fig. 4E and G. Figure 4E presents the time–frequency analysis of the glass collision signal, which reveals higher frequency components and a relatively short signal duration. In contrast, the clay collision signal (Fig. 4G) displays more pronounced low-frequency components and a longer duration. These distinctive spectral features provide a robust basis for classifying and distinguishing different impact conditions.

Building on this, we extracted over 10 time–frequency-related features for each impact type, with a minimum of 10 samples per condition. Using this dataset, we applied ensemble learning methods in combination with feature importance ranking to identify the most discriminative features. This enabled the development of a simple yet effective classification algorithm for recognizing different impact scenarios.

Figure 4H and I illustrates the classification results and feature importance rankings generated using the LightGBM algorithm [35] (an efficient ensemble learning method) and various time–frequency-related features. The confusion matrix in Fig. 4H demonstrates that the model effectively distinguished between 4 different impact conditions: none, steel, glass, and clay soil. This result highlights the suitability of the selected time–frequency features for improving the classification accuracy. The feature importance ranking (Fig. 4I) identified the peak voltage and Wilson amplitude as the most influential features. The average values of the top 3 features under different shocks are shown in Fig. S13. The effectiveness of these features for classification was further validated through a 2-dimensional feature distribution plot of peak voltage and Wilson amplitude (Fig. 4J), wherein different impact conditions formed distinct and well-separated regions. Thus, while peak voltage alone is insufficient to differentiate certain classes of signals, the inclusion of Wilson amplitude enables clear separation, significantly enhancing the classification and recognition accuracy.

Finally, Fig. 4K presents a decision flowchart for collision target recognition. By utilizing peak voltage and Wilson amplitude as core indicators, the flowchart outlines simple decision rules for progressively and accurately identifying the collision target. This lightweight, computationally efficient, and easily deployable algorithm offers strong support for reliable detection and classification of high-*g* shocks.

It is worth mentioning that in actual vehicle collision scenarios, the key to the collision sensor identifying the collision target lies in the amplitude and duration (width) of the collision signal at the moment of collision. The content of Fig. 4 verifies the proposed sensor’s ability to classify different types of shocks and its application potential in intelligent classification of automobile collisions. After being equipped in the vehicle and collecting datasets from actual collisions, the sensor is expected to be able to efficiently classify automobile collisions.

#### Development of self-powered microsystem for airbag control

**Fig. 5.**
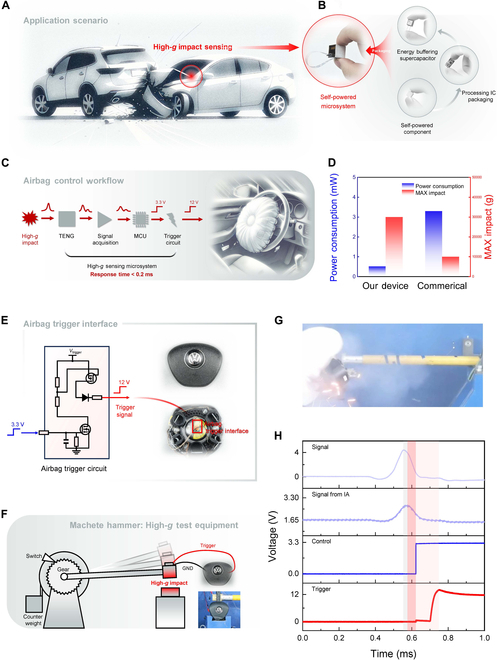
Self-powered microsystem for airbag deployment based on self-powered prestressed shock sensor. (A) Schematic of high-*g* shock sensing during a vehicle collision, illustrating the role of the sensor in capturing high-*g* impact signals. (B) Composition of the microsystem, including packaging, processing IC (integrated circuit) chips, and self-powered components, forming a complete detection and response unit. (C) Workflow diagram from high-*g* shock detection via the TENG, signal acquisition, microcontroller unit (<<MCU) signal processing, and triggering of the airbag deployment circuit. The total response time is under 0.2 ms. (D) Power consumption and maximum shock sensing capability of the self-powered microsystem and a commercial high-*g* sensor. The developed device significantly reduces power demand while withstanding higher shock intensities. (E) Airbag trigger interface, which converts a 3.3-V input to a 12-V output for airbag deployment in the steering wheel module. (F) Schematic and photograph of the “machete hammer” used for high-*g* testing. This device simulates high-*g* shocks to test sensor performance and reliability. (G) High-speed image capture during airbag deployment experiments, validating the functionality of the system under simulated collision scenarios. (H) Signal chain diagram for airbag deployment, including the initial signal, impact sensor signal, control signal, and final trigger signal. Red-shaded areas highlight the key timing points for airbag deployment, demonstrating the low-latency response of the system.

We developed a plug-and-play, self-powered microsystem designed for seamless integration into automotive airbag systems. Its performance was evaluated through simulated collision experiments, as depicted in Fig. 5. During a car collision, a high-*g* shock is generated (Fig. 5A), at which point the sensor must deliver a stable and high-amplitude voltage signal with minimal oscillation while also providing a reliable and shock-resistant power source for the airbag control and triggering circuitry.

To meet these requirements, we designed a compact self-powered microsystem (20.5 mm × 16 mm × 17 mm) that integrates the prestressed shock sensor, signal processing circuitry, and a backup power supply (Fig. 5B). The backend circuit, approximately fingertip-sized (20 mm × 13 mm × 3.6 mm), includes components for signal acquisition, control operations, and airbag triggering. The schematic diagrams of the microsystem’s internal structure and electrical connections are shown in Figs. S17 and S18. An 18-V stacked supercapacitor was selected to handle transient discharge demands during airbag activation. The system is compactly stacked, encapsulated in epoxy resin, and then housed in a stainless steel casing to improve the shock resistance of sensitive components and the overall system durability.

Figure 5C illustrates the operational workflow of the airbag control microsystem, detailing the process from the detection of high-*g* shock to the successful triggering of the airbag. The system achieves an ultra-fast response time of under 0.2 ms, far exceeding the human reaction time and common airbag deployment speeds. This high-speed response capability ensures reliable real-time performance and enhances safety in collision scenarios.

Figure 5D compares the performance of the prestressed shock sensor unit with that of a commercial high-*g* sensor in terms of power demand and maximum shock sensing capability. The self-powered microsystem demonstrates over 80% lower power demand and a shock measurement range approximately 3 times greater (see the Supplementary Materials). This enhanced performance results from its self-powered design, which eliminates the need for external power. Furthermore, the sensor exhibits minimal oscillation, eliminating the need for specialized filtering and modulation–demodulation circuitry. Instead, the system requires only a basic instrumentation amplifier for voltage extraction, significantly reducing power demand and the complexity of the peripheral circuitry (see Section S13 and Fig. S14). More detailed comparisons of the prestressed shock sensor and commercial shock sensors are shown in Table S2 and Figs. S19 and Fig. S20 in Section S17.

Figure 5E presents the control and triggering circuitry used for airbag deployment and its interface with the airbag system. To simulate collision scenarios safely, we employed the “machete hammer” testing device to generate transient high-*g* shocks (Fig. 5F). Upon impact, the self-powered shock sensor detected the impact and generated a response signal. Following the previously described workflow, the system successfully triggered the airbag (Fig. 5G), with the complete signal chain illustrated in Fig. 5H. The total response time of the microsystem, from shock detection to airbag activation, was approximately 0.15 ms, ensuring fast and reliable performance in real-time collision scenarios.

## Conclusion

This study presents a self-powered shock sensor based on a triboelectric transducer and a prestressed structure. By leveraging triboelectricity and the prestress mechanism, the sensor exhibits key advantages, including a high signal amplitude, low oscillation, and a reliable threshold-triggering effect under high-*g* accelerations reaching several thousand times the force of gravity. A multi-physics theoretical model and optimization framework were developed to refine the sensor design, resulting in an over 400% improvement in signal strength, attenuation ratio, and surface charge density. Impact tests at 10,000 × *g*, 20,000 × *g*, and 30,000 × *g* confirmed the excellent linearity and sensing performance of the sensor.

To extend the sensor to real-world automotive impacts, we developed a lightweight classification algorithm using ensemble learning and feature importance ranking to distinguish between hard (steel), brittle (glass), and soft (clay soil) collision targets. This algorithm addresses the complexity of potential target impacts in automobile collisions and demonstrates the potential for flexible deployment in low-power edge terminals.

Considering practical applications, we designed a highly integrated self-powered microsystem with a compact volume of less than 4.5 cm^3^. This microsystem, tailored for automotive collision scenarios, integrates a self-powered shock sensor, signal processing circuit, airbag triggering circuit, and shock-resistant backup power source. In simulated collision experiments, the microsystem has a total response time—from shock detection to airbag deployment—of under 0.2 ms, which is significantly faster than the human reaction time and conventional airbag deployment processes. Thus, the proposed microsystem effectively meets the requirements for real-time and reliable collision detection and airbag triggering.

The self-powered shock sensor, based on a prestressed triboelectric transducer, along with its integrated self-powered microsystem for airbag control, extends the application of triboelectric transducers to high-*g* impact environments. As such, our findings will aid the development of next-generation high-performance high-*g* shock sensors and sensing microsystems.

## Methods

### Device fabrication

The sensor devices were fabricated and assembled as follows (Fig. S15). First, we prepared 2 FR4 substrates with copper electrodes on both sides, serving as the core framework of the sensor. We also prepared 2 metal resin shells (top and bottom), silicone elastomer, and FEP triboelectric layers. The FR4 substrate and silicone elastomer were bonded to the inner surfaces of the resin shells using PUR gel. PUR gel has strong bonding and fast curing properties, enabling this step to be completed quickly. Next, we applied epoxy resin to the junction between the top and bottom parts, and the FEP triboelectric layer was placed between them. The assembled structure was clamped in place for 24 h to allow the epoxy resin to fully cure. The resulting device was then employed in subsequent testing.

The prepared device was subjected to multiple shocks and more than half a year of storage. It is worth noting that its response amplitude has almost no attenuation after these tests and storage, indicating its good stability (Fig. S16). It should be noted that due to the innovative purpose of this work and the limited test environment, the long-term effectiveness of the device after automotive-grade packaging in the actual vehicle needs to be tested separately before installation.

The microsystem assembly is relatively straightforward. A schematic of the internal structure is provided in Fig. S17. The microsystem is built around a custom high-density circuit system, which utilizes wafer-level packaging to minimize its volume. The microsystem integrates a high-voltage, high-energy-storage supercapacitor (Cellergy CLG18P002L12) and the prestressed sensor through a stacking method. These 2 components are electrically connected via metal wires and the high-density circuit board. All components within the microsystem are encapsulated in epoxy resin and protected by a high-strength, lightweight stainless steel shell to provide shock protection. The microsystem also includes pre-designated external interfaces to facilitate integration with external mechatronic systems.

### Experimental testing

The generation of high-*g* shocks under laboratory conditions is crucial for shock sensor testing. Simulated collision experiments allow multiple experiments to be performed efficiently without the need to replicate actual automotive collisions, thus reducing the testing time, logistical complications, and cost. Furthermore, they also help to validate the effectiveness and consistency of the theoretical model.

We employed a pre-calibrated “machete hammer” testing device (Nanjing University of Science and Technology) to generate instantaneous shock signals with accelerations of 10,000 × *g*, 20,000 × *g*, and 30,000 × *g*. In this setup, the sensor is fixed at the top end of the hammer, with its primary and reference electrodes connected to an oscilloscope (DSOX2024A, Keysight Technologies). Upon release, the hammerhead strikes the base, subjecting the sensor to intense shock forces. The voltage response of the sensor is recorded by the oscilloscope for analysis. Despite the limitations of the test equipment, we modified the Machete hammer device to generate a 3,000 × *g* shock to be closer to the vehicle collision environment. The relevant tests and analysis are shown in Fig. S21.

The airbag triggering experiments followed the same testing procedure. The supercapacitor within the microsystem was pre-charged. The airbag triggering and ground pins of the microsystem were connected to a real steering wheel airbag system. During the test, the supercapacitor powers the electronic system via an LDO (low dropout regulator) regulator (TPS7A26) while also providing power (>12 V) for airbag activation via the triggering circuit. When the sensor detects a high-*g* shock, the signal is passed through the instrumentation amplifier (AD8237) to the main control chip (STM32L476). Upon determining that the deployment conditions are met, the controller outputs a 3.3-V signal to activate the airbag triggering circuit.

## Data Availability

All data needed to support the conclusions in the paper are presented in the manuscript and the Supplementary Materials. Additional data related to this paper may be requested from the corresponding author upon request.
