# Supplementary material for: Self-Powered Microsystem for Ultra-Fast Crash Detection via Prestressed Triboelectric Sensing
Source: Research (Wash D C). 2025 Jul 2;8:0753. doi: 10.34133/research.0753 (PMC12218221; doi:10.34133/research.0753)
Supplement: Supplementary 1 — Supplementary Text Figs. S1 to S21 Tables S1 and S2 [file research.0753.f1.pdf]

Supplementary Materials

Self-Powered Microsystem for Ultra-Fast Crash Detection via Prestressed Triboelectric Sensing

Yiqun Wang [1], Yuhan Wang [1], Xinzhi Liu [1], Xiaofeng Wang [1, 3, *], Keren Dai [2, *], Zheng You [1, 3, *]

[1] *Department of Precision Instrument, Tsinghua University, Beijing, 100084, PR China*

[2] *School of Mechanical Engineering, Nanjing University of Science and Technology, Nanjing, 210094, PR China*

[3] *Beijing Advanced Innovation Center for Integrated Circuits, Tsinghua University, Beijing, 100084, PR China*

[*] Address correspondence to: Zheng You (yz-dpi@mail.tsinghua.edu.cn), Keren Dai (dkr@njust.edu.cn), and Xiaofeng Wang (xfw@mail.tsinghua.edu.cn)

1. Theoretical equations of the Multiphysics transient theoretical model 2
2. Comparison between the conventional TENG simulation and the transient simulation in this work 6
3. The stress distribution within the sensor calculated by the transient simulation 7
4. Comparison between the devices with and without prestress under 10,000 *g* shock 8
5. Voltage responses under different impacts (10,000/20,000/30,000 *g*) 9
6. Trend of the attenuation ratio under varying shock conditions 10
7. Trend of the attenuation ratio with different prestress 11
8. The average stress within the silicone elastomer with different prestress before the high-*g* shock 12
9. The stress mutation within the silicone elastomer with different prestresses due to the high-*g* shock 13
10. Relationship among surface charge density and prestress compression 14
11. The voltage response of the prestressed sensor exhibits a distinct impact threshold 15
12. Comparison of feature values under different shock conditions 16
13. Comparison between our sensing unit and commercial high-*g* sensor unit 17
14. Fabrication and assembly processes 21
15. Temporal stability test 22
16. Schematic diagram of the microsystem's internal structure and electrical connections 23
17. Comparison between this work and shock sensors in other principles 24
18. Device with lower prestress are used to sense medium-*g* shocks. 26

1. Theoretical equations of the Multiphysics transient theoretical model

1) Solid Mechanics

Linear Elastic Material:

$$\rho\frac{\partial^2 u}{\partial t^2} = \nabla\cdot(FS)^{\mathsf{T}} + F_{\mathrm{V}} \tag{S1}$$

$$\boldsymbol{F} = \boldsymbol{I} + \nabla\boldsymbol{u} \tag{S2}$$

$$S = S_{\mathrm{inel}} + S_{\mathrm{el}} \tag{S3}$$

$$\boldsymbol{\varepsilon}_{\mathrm{el}} = \frac{1}{2}\left(\boldsymbol{F}_{\mathrm{el}}^{\mathsf{T}}\boldsymbol{F}_{\boldsymbol{el}} - \boldsymbol{I}\right) \tag{S4}$$

$$\boldsymbol{F}_{\mathrm{el}} = \boldsymbol{F}\boldsymbol{F}_{\mathrm{inel}}^{\boldsymbol{-1}} \tag{S5}$$

$$S_{\mathrm{el}} = J_i F_{\mathrm{inel}}^{-1}(C:\varepsilon_{\mathrm{el}})F_{\mathrm{inel}}^{-\mathsf{T}} \tag{S6}$$

$$S_{\mathrm{inel}} = S_0 + S_{\mathrm{ext}} + S_{\mathrm{q}} \tag{S7}$$

$$\varepsilon = \frac{1}{2}[(\nabla u)^{\mathsf{T}} + \nabla u + (\nabla u)^{\mathsf{T}}\nabla u] \tag{S8}$$

$$C = C(\mathrm{E}, v) \tag{S9}$$

Contact:

$$T_n = \mathrm{if}(g_n \le 0, -p_n g_n, 0) \tag{S10}$$

$$p_n = f_p\frac{E_{\mathrm{char}}}{h_{\mathrm{min}}} \tag{S11}$$

Prescribed Displacement:

$$u_y = u_{0y} \tag{S12}$$

Fixed Constraint:

$$\boldsymbol{u} = 0 \tag{S13}$$

Gravity:

$$\boldsymbol{a}_f = -\boldsymbol{g} \tag{S14}$$

The theoretical modeling of elastic deformation induced by impact primarily involves the equations of solid mechanics as shown above.

ρ is the material density, u is the displacement field, t is time, $(FS)^{\mathrm{T}}$ represents the internal force influencing material motion, derived from the stress tensor S and deformation gradient tensor F, and F_{V} denotes the effect of external forces on the motion of the elastic body.This equation describes the dynamic behavior of materials in continuum mechanics.

$\boldsymbol{I}$ is the identity tensor, $\nabla\boldsymbol{u}$ is the displacement gradient tensor, and their sum yields the deformation gradient tensor $\boldsymbol{F}$。This equation is one of the fundamental equations in continuum mechanics for characterizing material deformation.

The stress tensor S can be decomposed into two components: the elastic part S_{el} and the inelastic part S_{inel} The inelastic part comprises the initial stress tensor S_0,external stress tensor S_{ext} and a stress tensor S_{q} associated with thermal, phase transformation, damage, or other inelastic processes. $\varepsilon_{\mathrm{el}}$ is the elastic strain tensor, $\boldsymbol{F}_{\mathrm{el}}$ is the elastic deformation gradient tensor, and $\boldsymbol{F}_{\mathrm{inel}}$ is the inelastic deformation gradient tensor.

J_{i} is the Jacobian determinant of elastic deformation, and C is the material's elasticity tensor, which can be determined from the Young's modulus E and Poisson's ratio v. ε is the strain tensor, quantifying the degree of deformation within the material.

In the contact equation, g_{n} represents the normal gap distance. When $g_{\mathrm{n}} \leq 0$, the contact pressure T_{n} equals the product of the contact pressure penalty factor p_{n} and the opposite number of the normal gap of the contact surface; otherwise, T_{n} equals 0.

E_{char} denotes the characteristic stiffness of the material, h_{min} is the minimum mesh size in the contact region, and f_{p} is the contact pressure penalty factor multiplier.

2) Electrostatics

Charge Conservation:

$$E = -\nabla V \tag{S15}$$

$$\nabla \cdot (\varepsilon_0 \varepsilon_{\mathrm{r}} E) = \rho_{\mathrm{v}} \tag{S16}$$

Zero Charge:

$$\boldsymbol{n} \cdot \boldsymbol{D} = 0 \tag{S17}$$

Surface Charge Density:

$$\boldsymbol{n} \cdot (\boldsymbol{D_1} - \boldsymbol{D_2}) = \rho_{\mathrm{s}} \tag{S18}$$

Grounding:

$$\boldsymbol{V} = 0 \tag{S19}$$

Terminal:

$$\int_{\partial\Omega} \boldsymbol{D} \cdot \boldsymbol{n}\, dS = Q_0 \tag{S20}$$

The theoretical modeling of electric field variations and electrode potential changes induced by the deformation of charged structures involves the equations of electrostatics as shown above.

E denotes the electric field intensity, V is the electric potential, ε_0 and ε_r are the vacuum and relative permittivity, respectively, ρ_v is the volume charge density, $\boldsymbol{n}$ is the surface normal vector, and $\boldsymbol{D}$ is the electric displacement vector. At the interface between two media, $\boldsymbol{D_1}$ and $\boldsymbol{D_2}$ represent the electric displacement vectors in the two media, and ρ_s is the surface charge density.

In the terminal charge equation, Q_0 is the total free charge surrounded by a closed surface of volume Ω. It states that the flux of the electric displacement vector $\boldsymbol{D}$ through the closed surface $\partial\Omega$ equals the total free charge Q_0 enclosed by that surface.

2. Comparison between the conventional quasi-static triboelectric nanogenerator simulation and the transient simulation in this work

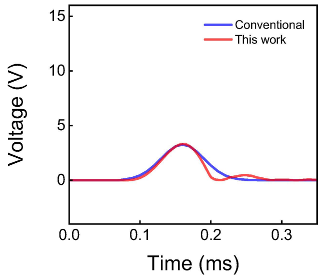

Fig. S1. Comparison between the conventional quasi-static simulation and the transient simulation proposed in this work.

	This work	Conventional TENG simulation
Type	Transient simulation	Static simulation
Input	Dynamic force/acceleration	Static displacement sweep
Multiphysics	Electrostatics and Solid Mechanics interacting via Moving Mesh	No coupling relationship, only electrostatic field
Movement	Various nonlinear irregular motions caused by force/acceleration	Linear motion generated by parametric sweeps
Space charge	Affected by the mechanical irregular deformation of the triboelectric layer	Linear distribution, only related to the displacement parameter
Output	Able to output the response waveform under shock	Can only output voltage value related to absolute displacement

Table S1. Comparison between the simulation method proposed in this work. and the conventional TENG simulation.

3. The stress distribution within the sensor calculated by the transient simulation

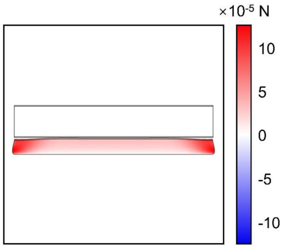

Fig. S2. Stress distribution derived from transient simulation.

4. Comparison between the simulation and experimental waveforms of the device without prestress under 10,000 *g* shock

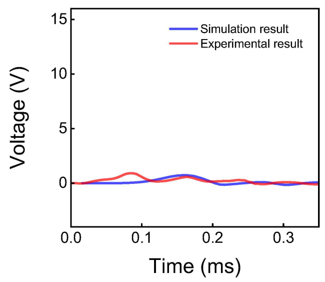

Fig. S3. Comparison between the simulation and experimental waveforms of the device without prestress under 10,000 *g* shock.

5. Voltage responses under different impacts (10,000/20,000/30,000 *g*)

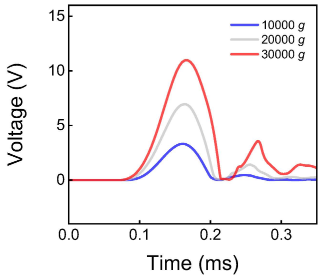

Fig. S4. Voltage response of the prestressed device under different impacts (10,000/20,000/30,000 *g*) calculated by the proposed simulation framework.

6. Trend of the attenuation ratio under varying shock conditions

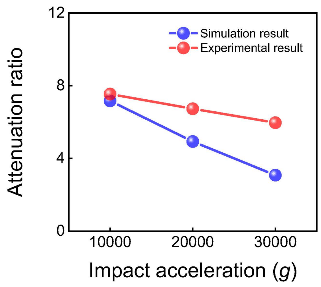

Fig. S5. Attenuation ratios for devices with different prestress compressions under varying gradient impacts (10,000/20,000/30,000 *g*): comparison between experimental results and simulations.

7. Trend of the attenuation ratio with different prestress

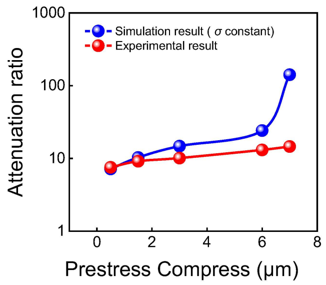

Fig. S6. Attenuation ratios for sensors with different prestress compressions under a given 10,000 *g* shock: comparison between experimental results and simulations.

8. The average stress within the silicone elastomer of sensors with different prestress compressions before the high-*g* shock

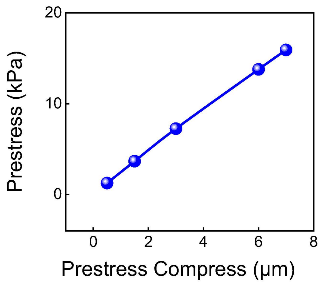

Fig. S7. Average internal stress within the silicone elastomer of sensors with different prestress compressions before impact, calculated using the proposed simulation framework.

9. The stress mutation within the silicone elastomer of devices with different prestresses due to the high-*g* shock

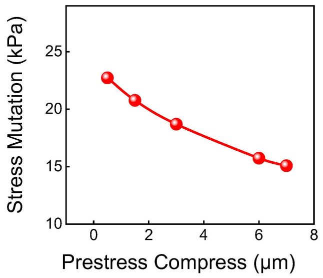

Fig. S8. Mutations in the average internal stress within the silicone elastomer with different prestress compressions caused by high-*g* shock, calculated using the proposed simulation framework.

10. Relationship among surface charge density and prestress compression

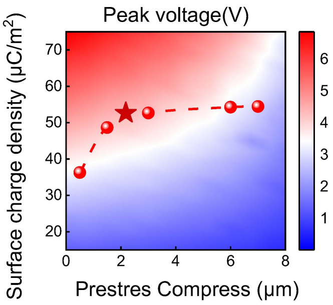

Fig. S9. Two-dimensional relationship among surface charge density, prestress displacement, and peak voltage, revealing the impact of prestress on enhancing surface charge density in the triboelectric layer.

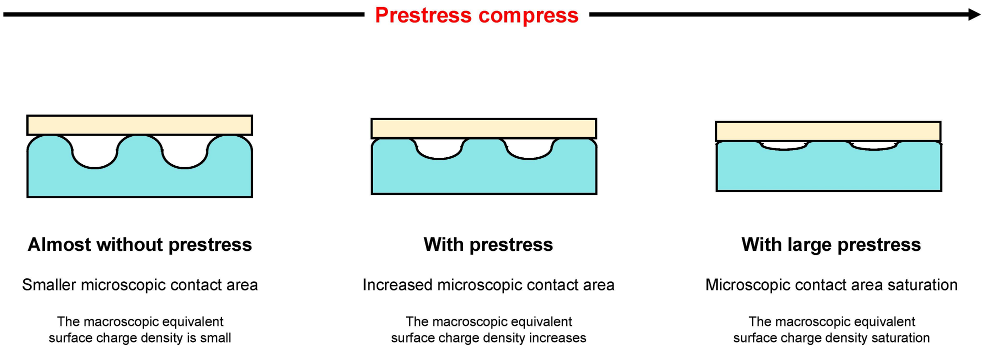

Fig. S10. Mechanism schematic: The essence of prestress compression enhancing surface charge density is the increase in the contact area at the microscopic level.

11. The voltage response of the prestressed sensor exhibits a distinct impact threshold

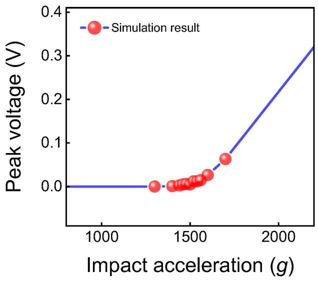

Fig. S11. Impact threshold scanning simulations of the optimized prestressed device based on the multi-physics theoretical model (the threshold is around 1500 *g*).

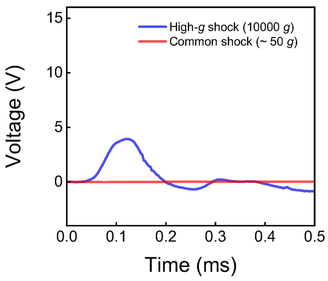

Fig. S12. Experiments have proven that the device will only trigger under high-g impacts, and will not trigger under normal collision impacts.

12. Comparison of feature values under different shock conditions

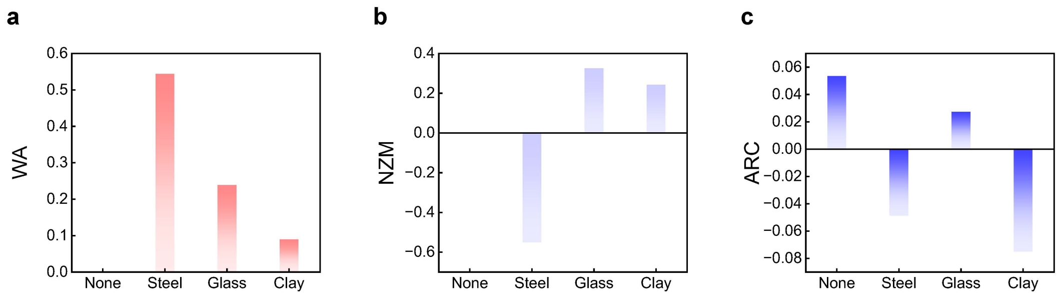

Fig. S13. Comparison of the top 3 feature values under different shock conditions. a. WA (Wilson Amplitude); b. NZM (Non-zero Median); c. ARC (Autoregressive Coefficient).

13. Comparison between the sensing unit composed of the prestressed sensor and basic peripheral circuitry with commercial high-*g* sensor unit

Power Consumption Comparison:

The total power consumption of each module in the ADXL001 adds up to approximately 2.5mA (including the timer, demodulation module, differential sensor, output amplifier, self-test module, and others). The TENG sensor's peripheral circuit only consumes about 212μA, which is primarily the power consumption of the instrumentation amplifier and the voltage divider resistors.

Internal Comparison:

TIMING GENERATOR

- **Function**: Provides the system clock signal to drive the internal modulation (MOD), demodulation (DEMOD), and differential sensor modules. The signal generated by the timing generator is typically used to control the device's sampling frequency, filtering functions, and synchronization timing.
- **Comparison**: The TENG directly generates a signal voltage, which is inherently related to the collision event. It does not require clock control for sampling, nor does it need modulation and demodulation processing, thus eliminating the need for the timing module.

MOD (Modulator)

- **Function**: Modulates the sensor's raw signal (usually a differential or low-frequency

signal) to a higher frequency band for subsequent processing and transmission, while minimizing the impact of low-frequency noise.

- **Comparison**: The TENG output is a high-amplitude, easily readable voltage signal, directly proportional to the collision intensity. No complex modulation processing is required.

DIFFERENTIAL SENSOR

- **Function**: The differential sensor is used to detect motion signals from the core part of the sensor, typically a precise acceleration detection unit (such as a MEMS structure). Its differential output reduces common-mode noise and improves signal quality.
- **Comparison**: The TENG directly perceives the external collision energy and generates a high-voltage signal, eliminating the need for a micro-mechanical acceleration detection process and the differential sensor module.

DEMOD AMP (Demodulation Amplifier)

- **Function**: Recovers the modulated signal to its original form and amplifies the signal's amplitude for subsequent signal processing or output.
- **Comparison**: The TENG directly outputs a high-voltage signal (3-4V), without needing modulation and demodulation processes, thus eliminating the corresponding demodulation amplifier module.

OUTPUT AMPLIFIER

- **Function**: Amplifies the sensor's signal to a level suitable for subsequent reading or processing, providing drive capability for loads or ADC interfaces.
- **Comparison**: The TENG amplifies the signal using a simple instrumentation amplifier, replacing the complex output amplifier module in the ADXL001.

SELF-TEST (Self-Test Module)

- **Function**: Provides an internal calibration function to test whether the sensor is operating normally. This is typically done by applying a known charge or signal to the sensor and verifying if the output response is correct.
- **Comparison**: The TENG's signal output is based on direct energy conversion from the collision. The working status can be determined by the signal's amplitude and its correspondence with external events, eliminating the need for a complex internal self-test circuit.

Comparative Advantages:

No Clock Module Needed:

- The TENG output is a high-amplitude voltage, directly related to the event, without the need for clock synchronization processing.
- Saves clock power consumption.

No Modulation-Demodulation Module Needed:

- The TENG directly outputs a signal with no low-frequency noise interference,

eliminating the need for a signal modulation chain.

- Simplifies the circuit and reduces power consumption.

No Complex Sensor Module Needed:

- The TENG is based on the energy harvesting principle, requiring no micro-mechanical or differential sensor units.
- Significant reduction in power consumption.

No Self-Test Module Needed:

- The TENG can verify the operational status through direct signal amplitude and event matching, without additional self-test circuitry.

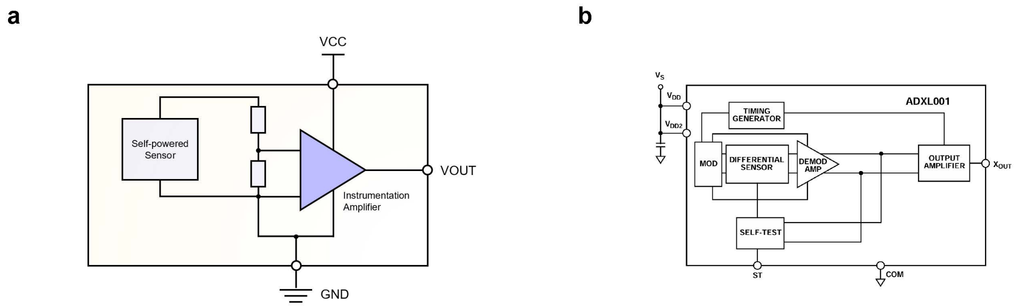

Fig. S14. Comparison of internal functional diagrams between the sensor proposed in this work and a commercial collision sensor. a. Our device purposed in this work; b. Commercial device (ADXL001).

14. Fabrication and assembly processes

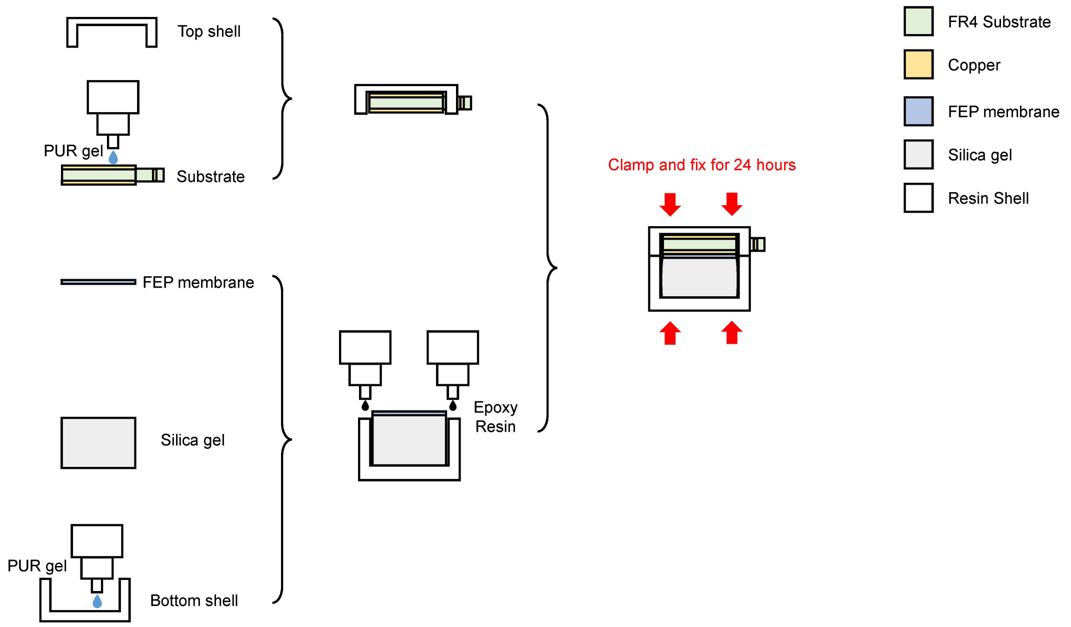

Fig. S15. Fabrication and assembly processes of the proposed sensor based on prestressed electrostatic transducer.

15. Temporal stability test

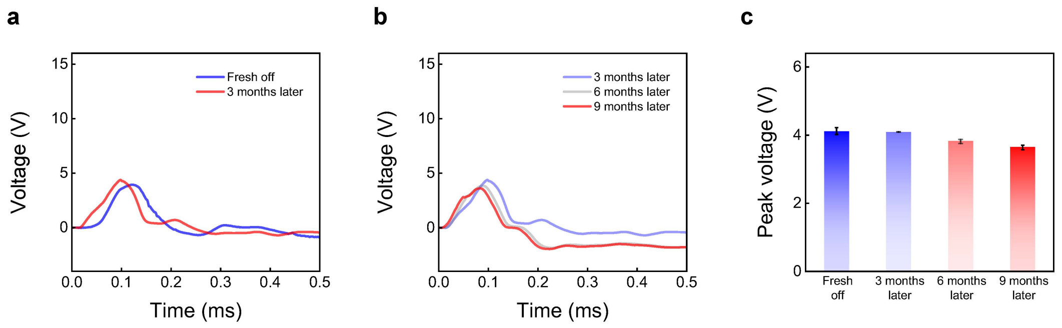

Fig. S16. The sensing performance of the device after three months of static storage. a. Comparison of the sensing waveforms under 10,000 *g* shock of the fresh off device and itself after three months of static storage; b. Comparison of the sensing waveforms under 10,000 *g* shock of the device after three/six/nine months of static storage; c. The influence of storage time on the device amplitude.

16. Schematic diagram of the microsystem's internal structure and electrical connections

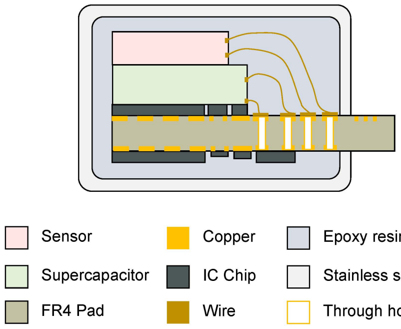

Fig. S17. Schematic diagram of the internal structure of the microsystem proposed in this work.

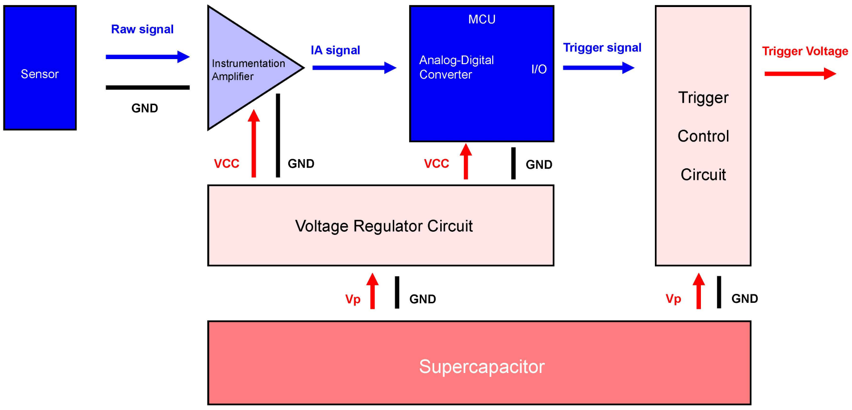

Fig. S18. Schematic diagram of the electrical connections of the microsystem proposed in this work.

17. Comparison between this work and shock sensors in other principles

	This work	Piezoelectric sensor	Capacitive sensor
Transducer principle	Electrostatic charge induction, direct voltage output	Piezoelectric effect, direct charge output into charge amplifier	Capacitance changes with structure, output based on power excitation
Sensing range	1500~30000 *g*	No more than 30000 *g*	No more than 500 *g*
Signal quality (>10000 *g*)	Large amplitude (>4V), large width (>200 us), small oscillation (Attenuation ratio>5)	Small amplitude (1V), moderate width (<100 us), severe oscillation (Duration>1ms)	Cannot withstand over 10000 *g*
Power supply	Self-powered and simple peripheral circuit	Device self-powers itself, while the peripheral circuits that require additional power are complex	Completely dependent on external power supply
Energy consumption	~0.5 mW	220 V supply	> 3 mW
Shape and size	Cuboid, 0.5 cm^3	Cylinder with large peripheral box	Cuboid, 0.5 cm^3

Table S2. Comparison between prestressed sensor of this work and shock sensors in other principles.

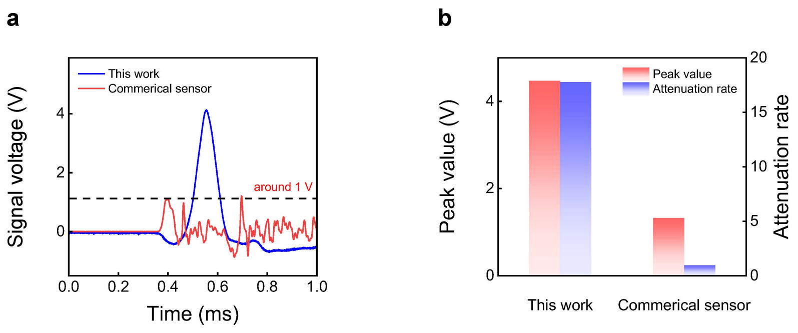

Fig. S19. Comparison of shock response signals of prestressed sensor in this and commercial shock sensor. a. Comparison of signal waveform; b. Comparison of peak value and attenuation ratio.

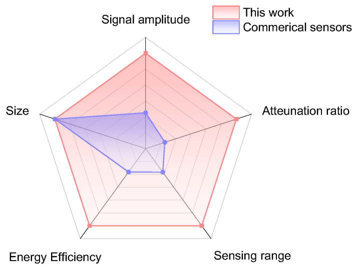

Fig. S20. The radar chart comparing the performance of the sensor proposed in this work with commercial sensors.

18. Device with lower prestress are used to sense medium-*g* shocks.

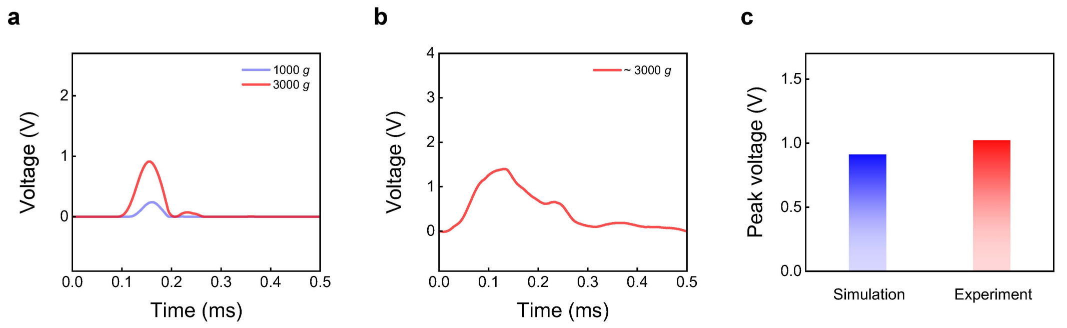

Fig. S21. Devices with lower prestress are used to sense medium-*g* shocks. a. The simulation results of device with lower prestress under 1000 and 3000 *g* shocks; b. The experimental result of device with lower prestress under 3000 *g* shock; c. The comparison of simulation rand experimental result of device with lower prestress under 3000 *g* shock.
